# Proteomic Analysis of the Extracellular Matrix in the Porcine Adrenal Cortex

**DOI:** 10.1021/acs.jproteome.5c00026

**Published:** 2025-06-10

**Authors:** Jean Lucas Kremer, Henrique Sanchez Ortega, Talita Souza-Siqueira, Claudia Blanes Angeli, Leo Kei Iwai, Giuseppe Palmisano, Claudimara Ferini Pacicco Lotfi

**Affiliations:** 1 Institute of Biomedical Sciences, Department of Anatomy, University of São Paulo, São Paulo, Av. Prof. Lineu Prestes, 2415, Butantã, São Paulo, SP 05508-000, Brazil; 2 Institute of Biomedical Sciences, Department of Parasitology, University of São Paulo, Av. Prof. Lineu Prestes, 1374, Butantã, São Paulo, SP 05508-000, Brazil; 3 School of Medicine, Department of Clinical Medicine, University of São Paulo, Av. Dr. Arnaldo, 455, Cerqueira César, São Paulo 01246903, Brazil; 4 Laboratory of Applied Toxinology, Center of Toxins, Immune-response and Cell Signaling LETA/CeTICS, Butantan Institute, Av. Vital Brasil, 1500, Butantã, São Paulo, SP 05503-900, Brazil; 5 School of Natural Science, Macquarie University, Angel Place, 123 Pitt Street, Sydney, NSW 2109, Australia

**Keywords:** extracellular matrix, adrenal gland, adrenal cortex, porcine

## Abstract

The extracellular matrix (ECM) is a complex network of proteins that provides structural support and influences tissue boundaries, biomechanical properties, and cell polarity. This study analyzed the ECM profile of the porcine adrenal cortex’s outer (OF = capsule + subcapsular + zona glomerulosa cells) and inner fractions (IF = zona fasciculata cells). Proteomic analysis of decellularized OF and IF samples identified 940 proteins, 27 collagens, 44 ECM glycoproteins, 9 proteoglycans, 20 ECM regulators, 5 ECM-associated proteins, 6 secreted factors, and 39 ECM candidates to compose the specific porcine adrenal matrisome. Among the ECM proteins identified, 113 are common to the OF and IF, while 16 were identified only in the OF and 21 in the IF. The analysis of protein abundance differences showed three proteins (Lamc1, Perlecan, and Tgm2) significantly abundant in OF compared to IF. In IF, 11 proteins (Col1α1, Col1α2, Col6α1, Col6α2, Col6α3, Col6α6, Col14α1, Ecm1, Fga, Fgb, and Fgg) were more abundant than OF. The comparative analysis of the quantification ECM proteins from the decellularized adrenal cortex of rats, humans, and pigs showed that in porcine samples, the ECM-quantified proteins are more abundant in the IF, while in rats and human samples, the more abundant ECM-quantified proteins are in the OF. These findings provide valuable insights into the potential of using pigs as a biomedical model and an essential tool for translational medical research.

## Graphical abstract

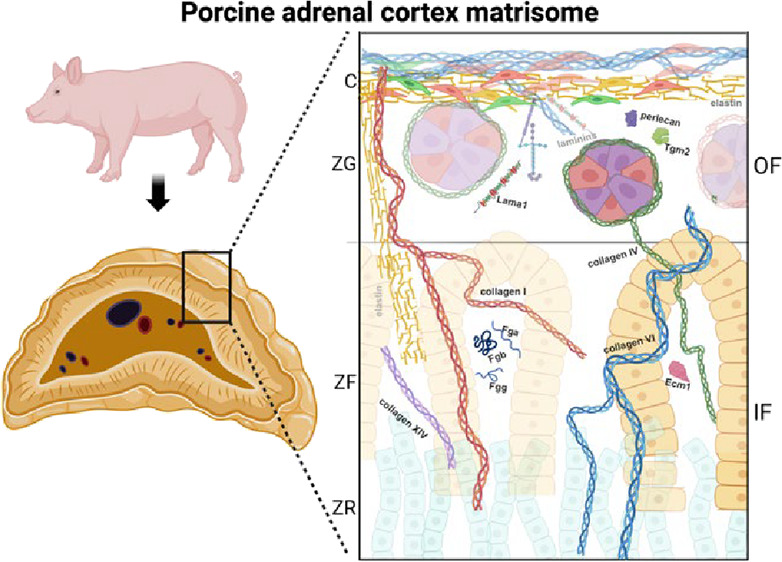

## Introduction

The extracellular matrix (ECM), a complex meshwork of proteins, provides a three-dimensional architectural scaffold that defines tissue boundaries and determines biomechanical properties and cell polarity.^1,2^ It serves as an adhesive substrate supporting cell migration and growth factor binding. ECM proteins help surface receptors interpret biochemical signals that regulate cell survival, proliferation, differentiation, and stemness.^3,4^ Its role has become recognized as crucial to overall health and disease, leading to several clinical trials to modify the ECM.^5,6^

Recently, we reported on the ECM protein profile of the adrenal cortex of Sprague–Dawley rats and humans.^7,8^ The adrenal gland consists of a cortex and a medulla. The adrenal cortex consists of distinct zones with specialized steroid-producing functions: the zona glomerulosa (ZG), zona fasciculata (ZF), and zona reticularis (ZR), which are responsible for the production of mineralocorticoids, glucocorticoids, and androgens, respectively.^9,10^ The functional heterogeneity of these zones stems from the complex interplay between endocrine and paracrine signaling mechanisms, along with the intricate and not well-understood organization and composition of the ECM.^11^ Research on the ECM in rat and bovine adrenal cortices has highlighted its possible role in regulating adrenocortical steroidogenesis, cell proliferation, and tissue remodeling.^12^ In our report of ECM proteomic profiles of rats and humans of decellularized outer and inner fraction samples of the adrenal gland, 32 proteins were classified in rats as ECM-component and ECM-related. In humans, 121 ECM proteins were identified and categorized into glycoproteins, collagens, ECM regulators, proteoglycans, ECM-affiliated proteins, and secreted factors.^7,8^ Here, we report an analogous study of adrenal cortex ECM protein identification in the model organism , the domestic pig. Pigs share similarities with humans in anatomical size and structure, genome, physiology, and immunology, making them preferred animals for human xenotransplantation as a potential alternative approach for treating adrenal insufficiency.^13,14^ Porcine adrenals have three adrenal zones, and most steroidogenic enzymes exist in humans,^15^ while in murine, the ZR is not recognized.^9,10^ In pigs, cortisol and aldosterone biosynthesis is catalyzed by a single enzyme, cytochrome P450 11β-hydroxylase (CYP11B), expressed throughout the adrenal cortex.^16,17^

While rat and human adrenal cortex ECM-core proteins and ECM-associated proteins have been identified,^7,8^ the precise composition, spatial distribution, and functional relevance of porcine ECM components in the adrenal cortex have yet to be fully elucidated. This work aims to identify the constitution of the ECM of the porcine adrenal cortex. We compared OF and IF proteomic profiles to identify the ECM composition of the outer (OF = capsule + ZG) and inner fractions (IF = ZF + ZR) obtained from dissected and decellularized porcine adrenal glands. Some findings were validated by histochemistry and immunohistochemistry, and the relevance of the observed differentially expressed ECM and ECM-associated proteins of the OF and IF was evaluated. Finally, these findings may contribute to understanding the potential of pigs as a biomedical model and an important tool for translational medical research.

## Experimental Section

### Animals

Adrenal glands from five adult male and female porcines were collected from the experimental surgery unit of the Laboratory of Anesthesiology at the University of São Paulo’s School of Medicine. The study was approved by the Animal Experimentation Ethics Committee of both the School of Medicine and the Institute of Biomedical Sciences (protocols 1708/2021 and 4404220321).

### Obtaining Samples of the Outer and Inner Fractions of the Porcine Adrenal Cortex: Sample Decellularization and Preparation

Kremer et al.^8^ provided a detailed description of obtaining samples from the outer and inner fractions of the porcine adrenal cortex as well as the decellularization protocol. Remaining intact cells were verified by DNA quantification in NanoDrop (Thermo Fisher Scientific) and considered sufficient at a concentration <50 ng/μL (Figure S1A). Decellularized samples of adrenal and control tissues were stained with 4 mg/mL 4′,6-diamidino-2-phenylindole (DAPI, Sigma-Aldrich, Massachusetts, USA) and analyzed under a fluorescence microscope (Nikon) (Figure S1B). The list of peptides for which proteins were identified can be found in Table S1.

### Data, Bioinformatics, and Statistical Analysis

The raw files were processed as described by Kremer et al.^8^ using MaxQuant software version 2.1.4 with Andromeda as a search engine against databases (46,174 entries, downloaded from Uniprot.org in March 2024) and normalized using the "normalize.quantiles" method from the R package preprocessCore, as outlined by Srivastava et al.^18^ The total identified and regulated proteins were compared using available matrisome databases^19^ for and (https://sites.google.com/uic.edu/matrisome/matrisome-annotations/homo-sapiens; https://sites.google.com/uic.edu/matrisome/matrisome-annotations/mus-musculus), accessed in March 2024. The mass spectrometry proteomics data have been deposited to the ProteomeXchange Consortium via PRIDE with the data set identifier PXD055486 and 10.6019/PXD055486.

### Histochemistry and Immunohistochemistry

The porcine adrenal samples underwent immunohistochemical and histochemical techniques, as described by Kremer et al.^7^ Representative hematoxylin and eosin (H&E) staining images are presented in the Supporting Information (Figure S2). For immunohistochemistry, an anticollagen VI antibody (1:200; Abcam Ab182744) was used alongside secondary antibodies, specifically Alexa Fluor 594 antirabbit IgG fluorescent antibody (1:2000; Jackson ImmunoResearch Laboratories, Pennsylvania, USA).

## Results and Discussion

### Quantitative Proteomic Analysis of Decellularized Porcine Adrenal Gland

The decellularized samples of the outer fraction (OF = capsule + ZG) and inner fraction (IF = ZF + ZR) from porcine adrenal glands were analyzed by using label-free quantitative (LFQ) proteomics and validated by histochemistry and immunohistochemistry. The proteomic data analysis workflow is shown in Figure 1, which shows porcine adrenal glands fractionated into an outer and an inner fraction. ECM enrichment was accomplished via decellularization. The extracted proteins were subsequently prepared, purified, and analyzed using LFQ proteomics based on liquid chromatography–mass spectrometry (LC–MS/MS).

**Figure 1:**
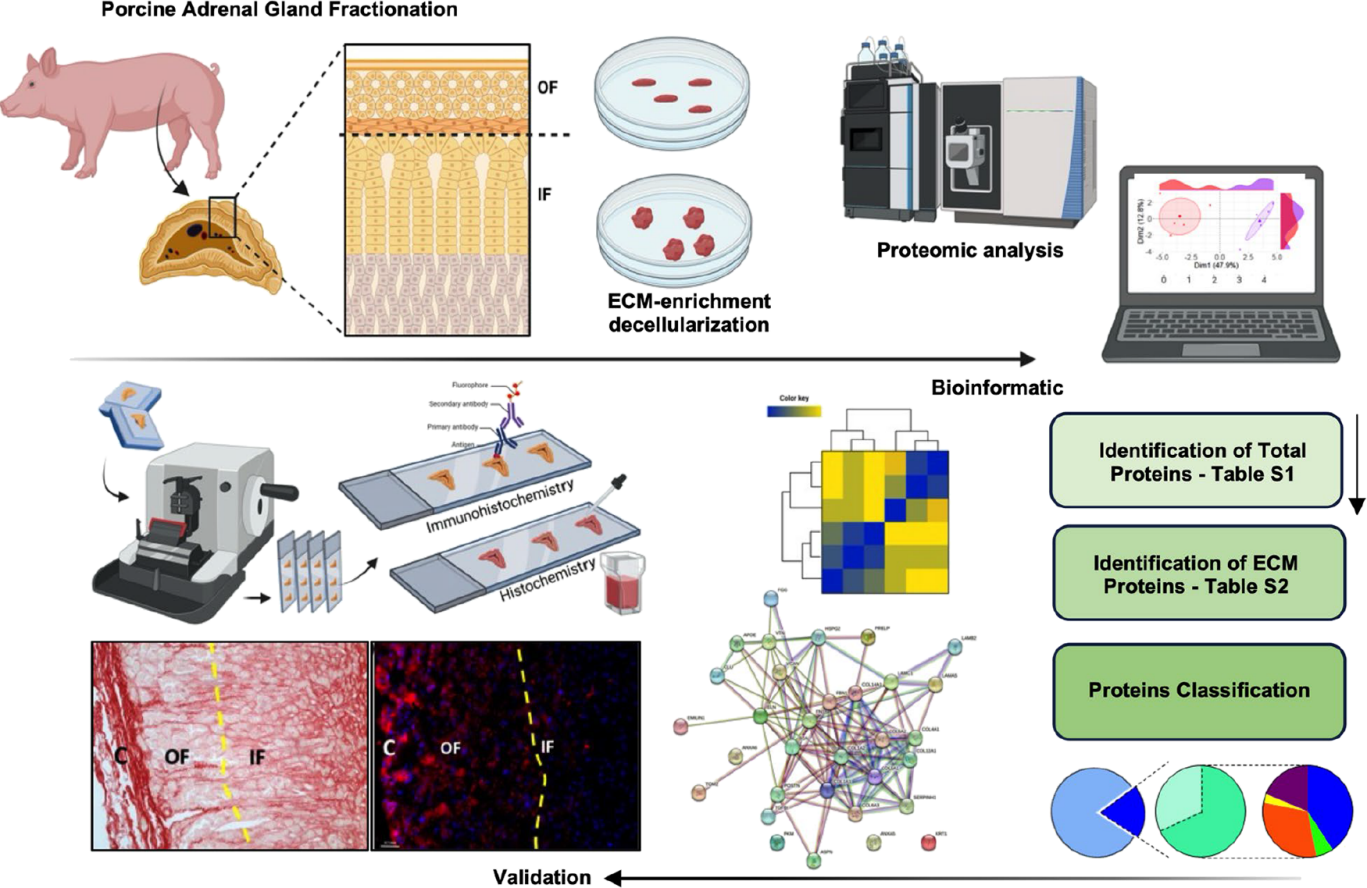
Schematic overview illustrates the methodology applied across the different phases of the study. Porcine adrenal glands (*n* = 5) were collected and separated into an outer fraction (OF) and an inner fraction (IF). The extracellular matrix (ECM) was enriched and prepared for proteomic analysis. High-resolution, proteome-wide semiquantitative LC-MS/MS was used to quantify ECM proteins, followed by bioinformatic analysis using Perseus software. Protein identification requires at least 60% of valid values in one or more groups. ECM proteins were categorized, and protein–protein interaction networks and functional enrichment analyses were generated using the STRING database tool (https://string-db.org). A Student’s *t* test (*p* < 0.05) was employed to identify significantly regulated proteins between OF and IF fractions. Validation steps included cross-referencing matrisome databases and conducting histochemical and immunohistochemical analyses.

The principal component analysis (PCA) with spectral decomposition highlights the ability of ECM protein stratification to distinguish between OF and IF groups (Figure 2A). A plot was generated using R software to visualize the distribution of proteins across the 10 dimensions analyzed in PCA. All dimensional analyses and contributions are represented in Figure S3. The dimensional contributions of each protein (dimensions 1 and 2) suggest a specific regulation and signature of fraction samples (Figure 2B). In total, 940 proteins were identified (Table S2), with 150 categorized as ECM proteins by Gene Ontology (Figure 2C). We used a combination of human and mouse matrisome databases, protein annotations using UniProtKB codes (https://www.uniprot.org/), and Gene Ontology AmiGO2 (https://amigo.geneontology.org/amigo/landing), through which 111 ECM proteins were validated. In comparison, 39 ECM proteins were not found in the matrisome databases used (Figure 2D).

**Figure 2:**
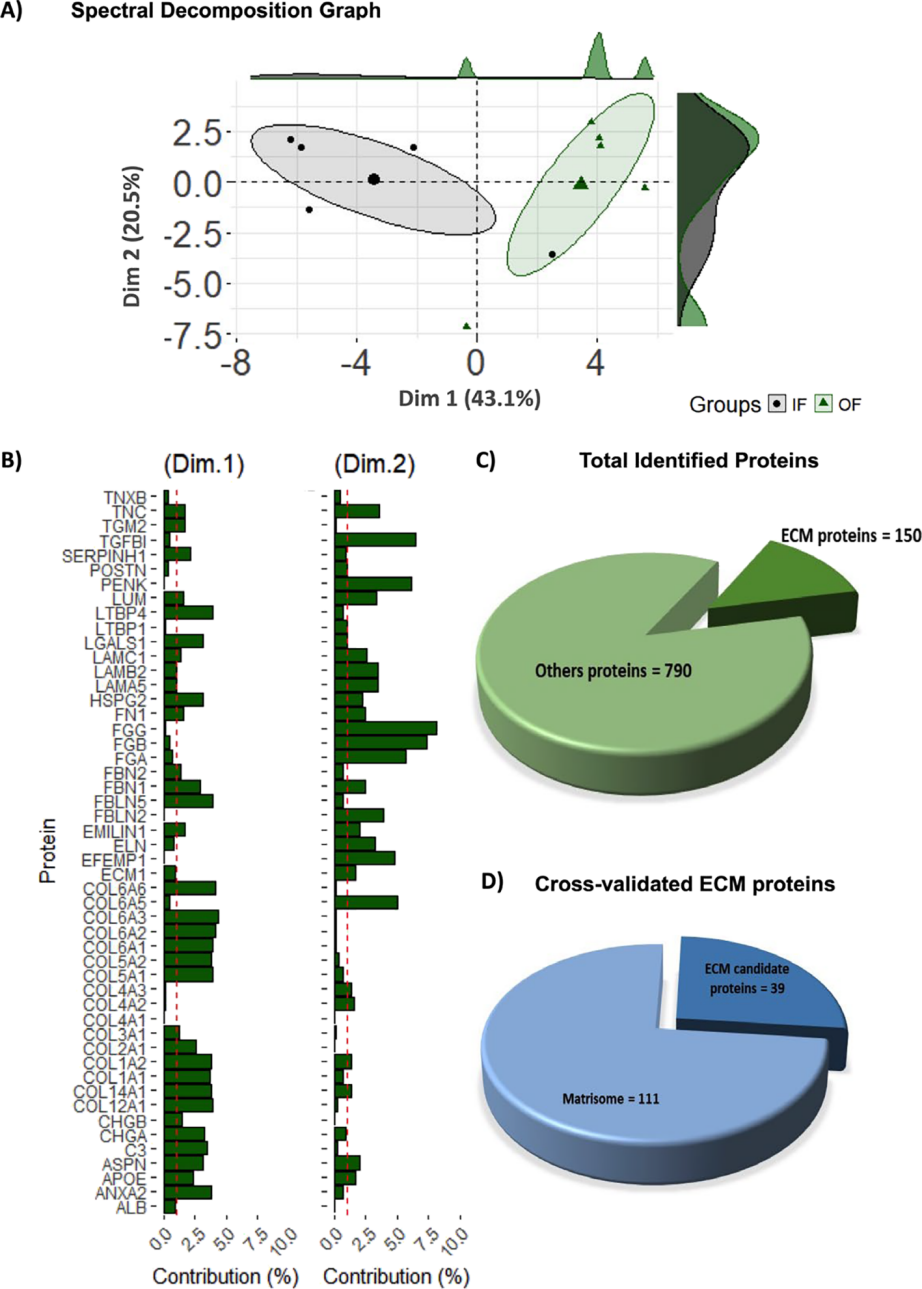
Proteomic analysis of porcine adrenal samples and cross-validation of adrenal ECM proteins. (A) Principal component analysis (PCA) using spectral decomposition demonstrates that stratification of the extracellular matrix (ECM) proteins effectively distinguishes between outer fraction (OF) and inner fraction (IF) samples. (B) PCA biplot showing the contribution of each protein to dimensions 1 and 2. (C) Overview of the total and ECM-specific proteins identified. (D) ECM proteins cross-validated in human and mouse matrisomes and ECM proteins not identified matrisome databases, selected according to gene ontology (GO) to be included in the swine adrenal cortex matrisome.

The ECM proteins identified were categorized as collagen (27), glycoproteins (44), proteoglycans (9), ECM regulatory proteins (20), ECM-associated proteins (5), secreted factors (6), and other ECM proteins (39). These 39 ECM proteins are candidates to compose the specific porcine adrenal matrisome (Figure 3A). Of the ECM proteins identified, 113 are common to the OF and IF, while 16 were identified only in the OF and 21 in the IF (Figure 3B and Table S3). Of the 16 OF proteins validated on matrisome databases, 2 proteins are collagens (collagen type XXVIII alpha 1 chain and collagen type VIII alpha 1 chain), 2 are ECM glycoproteins (adipocyte-binding protein 1 and cartilage intermediate layer protein), 2 are ECM regulators (serpinc1/antithrombin-III and serpina3-2/alfa-1-antiquimotripsina 2), 1 is ECM-associated protein (ficolin-2), and 9 are ECM candidates to compose the porcine adrenal matrisome.

**Figure 3:**
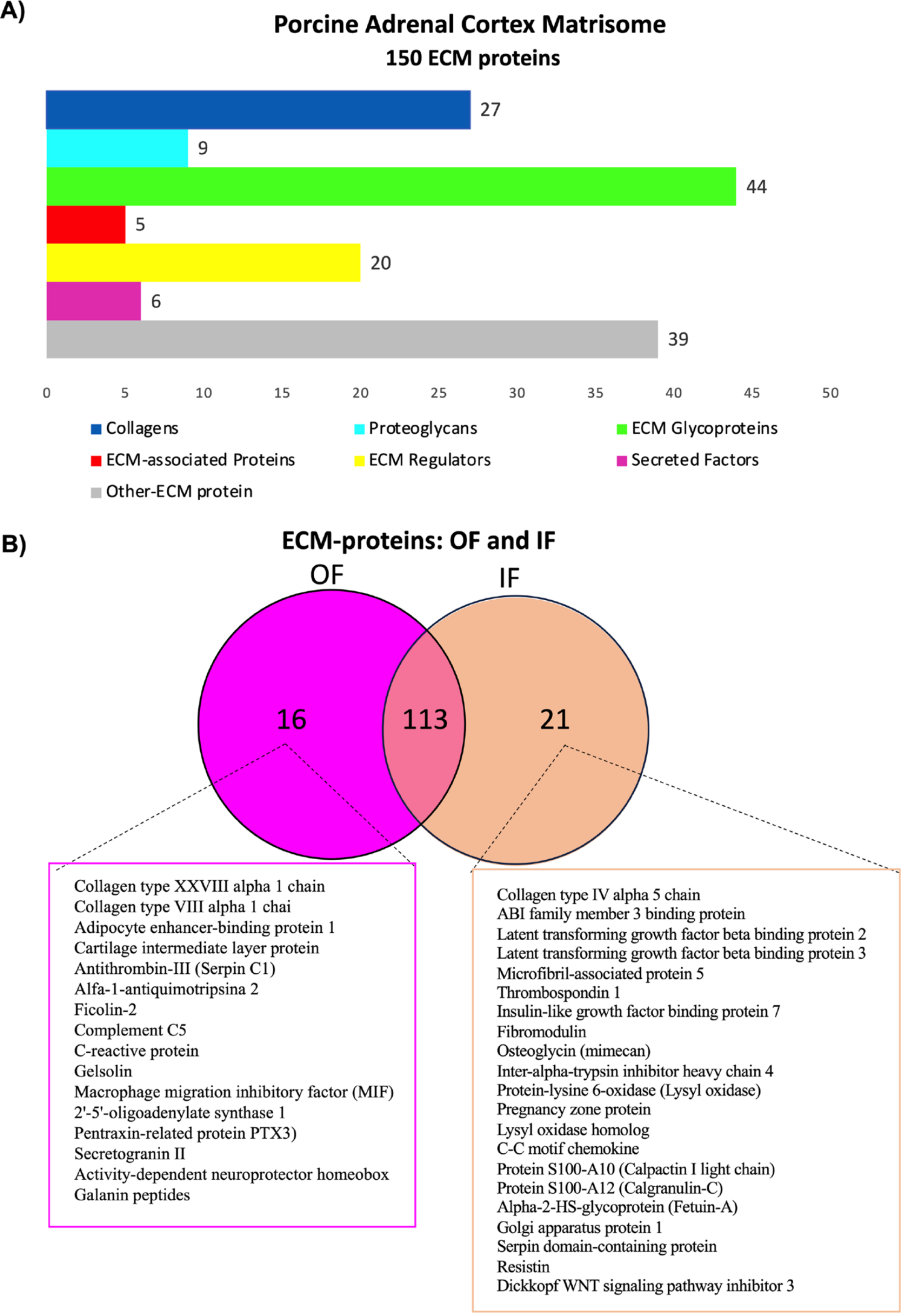
ECM-protein categorizations and identification in OF and IF fractions. (A) ECM-protein categorization: blue, collagens; cyan blue, proteoglycans; green, ECM glycoproteins; red, ECM-associated protein; yellow, ECM regulators; pink, secreted factors; gray, other ECM proteins. (B) ECM proteins are common between OF and IF fractions and are identified only in the OF or IF.

Of the 21 IF proteins validated, one is collagen (collagen type IV alpha 5 chain), 6 are ECM glycoproteins (ABI family member 3 binding protein; latent transforming growth factor beta binding protein 2 and 3; microfibril-associated protein 5; thrombospondin 1; and insulin-like growth factor binding protein 7), 2 are proteoglycans (fibromodulin and osteoglycin/mimecan), 4 are ECM regulators (interalpha-trypsin inhibitor heavy chain 4; lysyl-oxidase; pregnancy zone protein; and lysyl-oxidase homologue), and 3 are secreted factors (C–C motif chemokine, protein S100-A10/calpactin I light chain, and protein S100-A12/calgranulin-C). Five proteins are ECM candidates to compose the porcine adrenal matrisome (Figure 3B and Table S3).

Of the two collagens (XXVIII and VIII alpha 1 chain) identified in the OF, collagen XXVIII, which forms beaded filaments, is the most intriguing since, in mice, it has been shown to restrict tissue distribution in the peripheral nervous system.^20^ In the published draft map of the human proteome, the Col28A1 gene was highly expressed in the adult spinal cord and weakly expressed in various organs, including the adult adrenal gland.^21^ Moreover, a very restricted collagen XXVIII expression was detected in BM around the murine lung vessels, airways, and alveoli.^22^ Interestingly, collagen XXVIII was not identified in the OF ECM of humans or rat decellularized adrenal cortex.^7,8^ Although collagen XXVIII shares some similarities with collagens VI and VII, studies from zebrafish and mice indicate that this collagen is unique in evolution, expression, and structure.^23^ Also, its expression may be broader and involve repair processes.^22^

Collagen VIII is a network-forming collagen, like collagen IV and collagen X.^2^ Collagen VIII is a nonfibrillar collagen that forms a complex hexagonal network deposited at various highly specialized matrices, such as vascular walls and the Descemet membrane of the eye. Various cell types, such as endothelial cells, vascular smooth muscle cells, mast cells, monocytes, macrophages, and T cells, synthesize it.^24^ Collagen VIII can give rise to matricryptins, bioactive fragments released by proteolytic cleavage of collagens that regulate several physiological and pathological processes such as development, angiogenesis, tissue repair, tumor growth, and metastasis.^25^ Like collagen XXVIII, collagen VIII has not been identified in the decellularized adrenal cortex of rats and humans.^7,8^

One collagen type was identified only in porcine IF, the collagen type IV alpha 5 chain. In rats, the collagen type IV, alpha 1, and 2 chains are more abundant in the IF than in the OF but are present in both.^7,26^ In human fetal adrenal glands and the decellularized IF of the human adrenal cortex, collagen IV is expressed throughout the adrenal gland.^27,8^ In vitro studies on fetal primary adrenal cultures show collagen IV promotes hydroxy-delta-5-steroid dehydrogenase (HSD3B2) expression and cortisol production.^27^ Therefore, collagen IV seems to be involved in adrenal cortex steroidogenesis, particularly cortisol production, in the three mammalian species studied: rat, porcine, and human.

### ECM Regulation of the Outer and Inner Fractions of the Porcine Adrenal Cortex

Using the LFQ method, 50 ECM proteins were quantified (Figure 4). The comparative analysis of ECM protein quantification by LFQ of ECM proteins from decellularized OF and IF adrenal cortex of rats, humans, and pigs showed a distinct characteristic.

**Figure 4:**
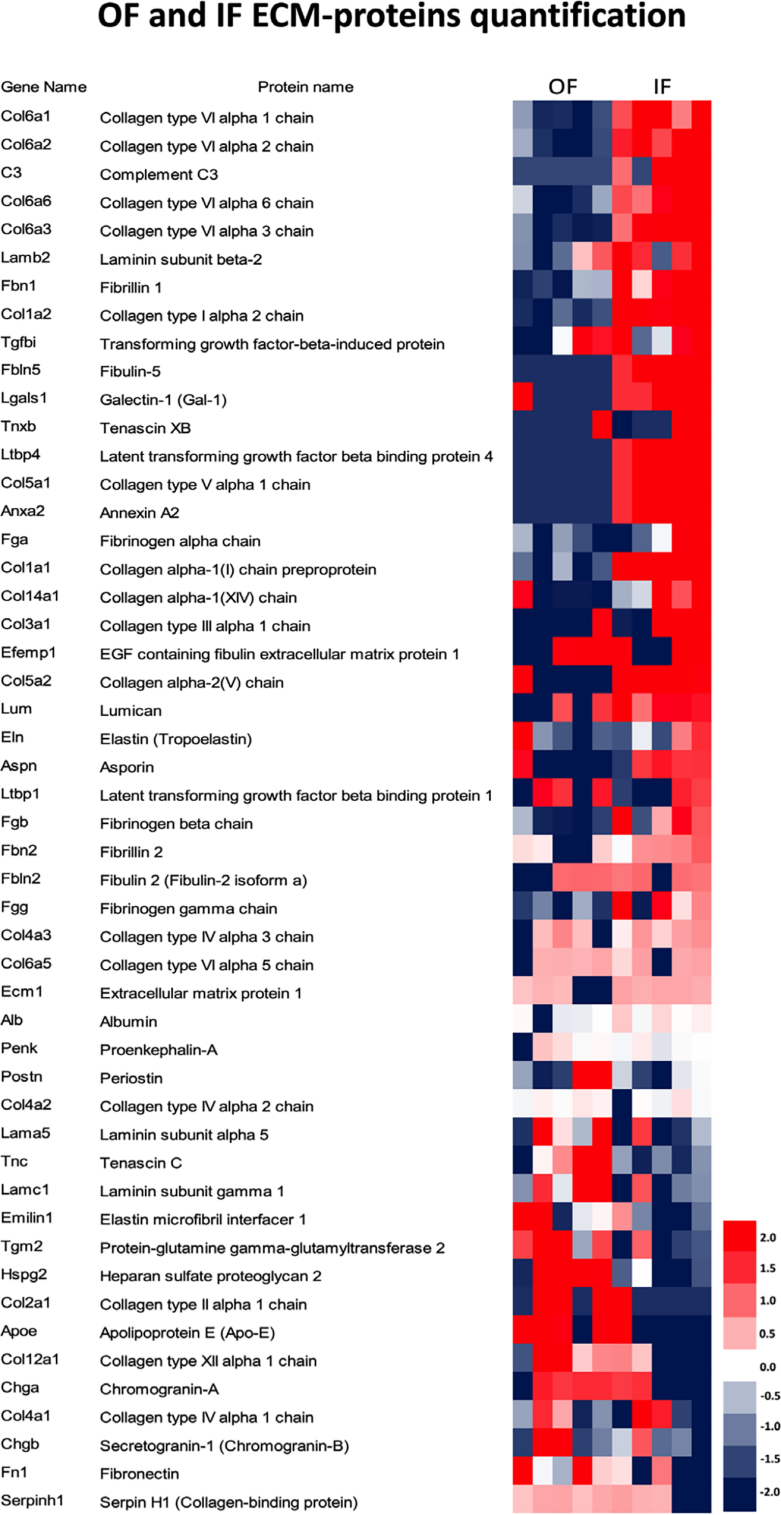
ECM proteins exhibit differential expression between the outer and inner fractions of the porcine adrenal cortex. Heatmap showing the differentially expressed ECM proteins in the outer (OF) and inner (IF) cortex fractions. The analysis was based on *z*-scores derived from label-free quantification (LFQ) intensity values, with red indicating up-regulated proteins and blue indicating downregulated proteins.

While rat and human samples have shown that the ECM-quantified proteins are more abundant in the OF,^7,8^ in porcine samples, the more abundant ECM-quantified proteins are in the IF.

The analysis of protein abundance differences (Figure 5) showed three proteins significantly abundant in the OF compared to the IF: laminin subunit gamma 1 (Lamc1), heparan sulfate proteoglycan 2-Perlecan (Hspg2), and transglutaminase 2 (Tgm2). Laminins are glycoproteins found in the basement membrane (BM) and are fundamental in cell adhesion, migration, and signaling.^28^ Laminins exhibited a zonal distribution in both adult and fetal human adrenal cortex^29,30^ and were found to influence adrenal cortex function in rats and humans. In cell culture studies using rat and human fetal adrenal cortex, laminin inhibited steroidogenesis while promoting cell proliferation.^27^ Although the laminin subunit gamma has been significantly abundant in OF, all laminin chains, alpha, beta, and gamma have been identified in the porcine adrenal cortex (Table S2). Perlecan, a BM component, stabilizes the ECM.^31^ Like what was found in the proteomic analysis of the human adrenal,^8^ perlecan is more abundant in the porcine OF of the adrenal gland, providing peculiar characteristics to this outermost fraction of the adrenal cortex. Tmg2 was selected as the ECM-regulator component in a proteomic characterization of stem cell-derived extracellular matrices^32^ and located in collagen-containing ECM.^33^ It would be interesting to test whether the abundance of the Tmg2 protein is associated with the cell renewal played by the adrenal cortex OF since both capsular cells and ZG are considered the progenitor niche of adrenocortical cells.^34,35^

**Figure 5:**
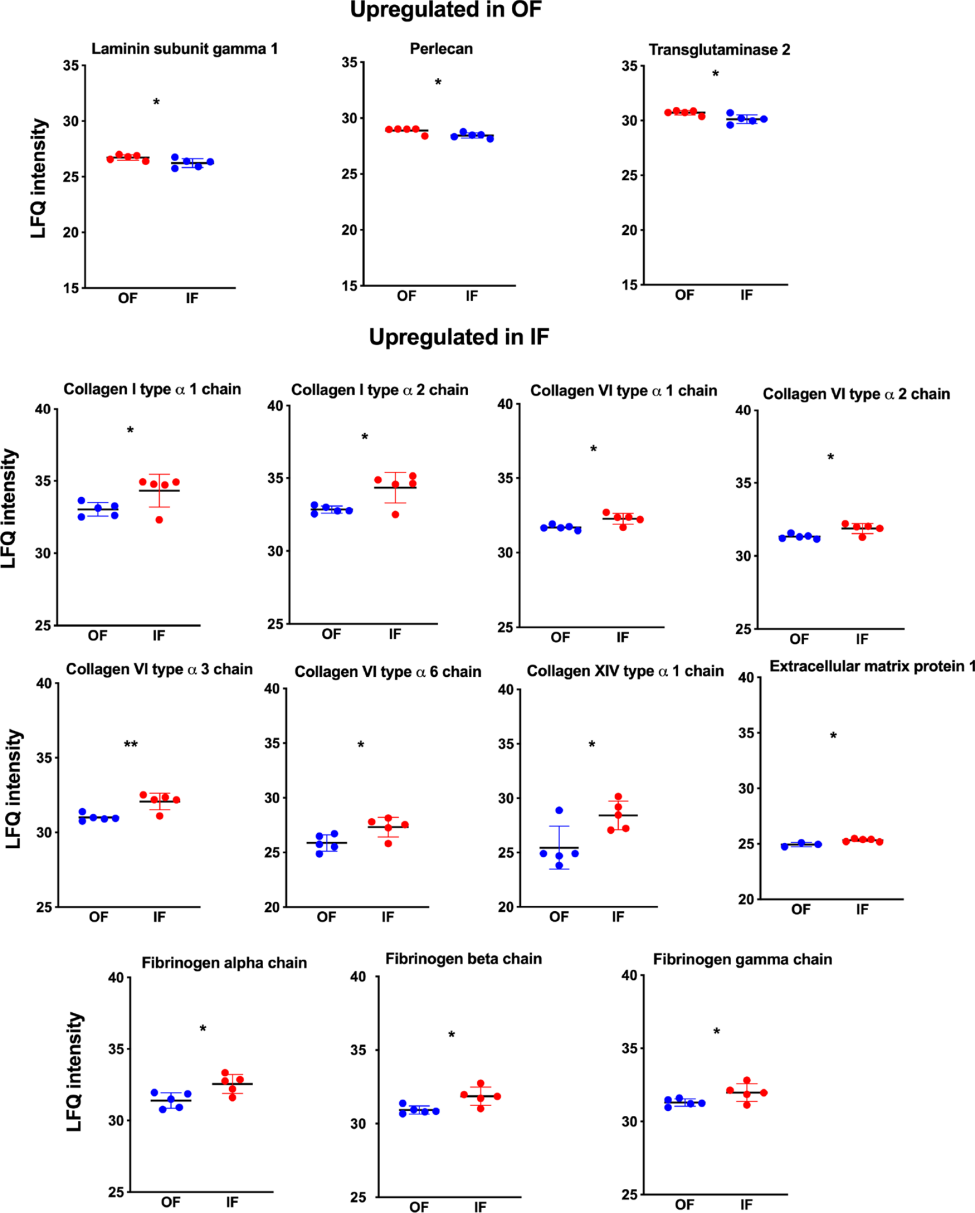
Up-regulated ECM proteins in outer and inner fractions of the porcine adrenal cortex. Proteins were identified as differentially regulated in the outer fraction of OF (red) and the inner fraction of IF (blue) by proteomic analysis. Student test: *p ≤ 0.05, ** *p* ≤ 0.01. Each dot represents a sample’s label-free quantification (LFQ) value, while the horizontal line indicates the mean LFQ value ± standard deviation (SD).

In IF, 11 proteins (collagen type I alpha 1 chain (Col1α1), collagen type I alpha 2 chain (Col1α2), collagen type VI alpha 1 chain (Col6α1), collagen type VI alpha 2 chain (Col6α2), collagen type VI alpha3 chain (Col6α3), collagen type VI alpha 6 chain (Col6α6), collagen type XIV alpha 1 chain (Col14α1), extracellular matrix protein 1 (Ecm1), fibrinogen alpha chain (Fga), fibrinogen beta chain (Fgb), and fibrinogen gamma chain (Fgg) were more abundant compared to OF (Figure 5).

Col1α1 and Col1α2 are fibrillar collagens that contribute to mechanical strength and tissue stability. They interact with collagens IV, V, VI, XII, and XIV, as well as with fibronectin and proteoglycans, which regulate cell proliferation and differentiation.^25^ Col VI provides mechanical support and specific cytoprotective functions, including inhibiting apoptosis and oxidative stress, while also influencing cell differentiation and autophagic processes.^36^ Interestingly, in contrast to what was observed in porcine adrenal IF, in the rat and human adrenal cortex, the greatest abundance of collagens occurs in the OF.^7,8^

Fibrinogen (Fg) plays a primary role in clot formation. After being converted to fibrin by thrombin, it binds to various compounds, particularly growth factors, making Fg a player in ECM physiology.^37^ Fg deposition changes the topology of the ECM, providing a surface for cell migration and matrix remodeling.^38^ Based on the centripetal migration hypothesis of the adrenocortical renewal process,^34,39^ testing the role and importance of Fb abundance of IF in cell migration and differentiation in the adrenal cortex would be interesting.

Histochemistry and immunohistochemistry confirmed that Col I is more abundant in IF than in OF (Figure 6A) and that collagen VI (Col6) is significantly more abundant in IF than in OF (Figure 6B).

**Figure 6:**
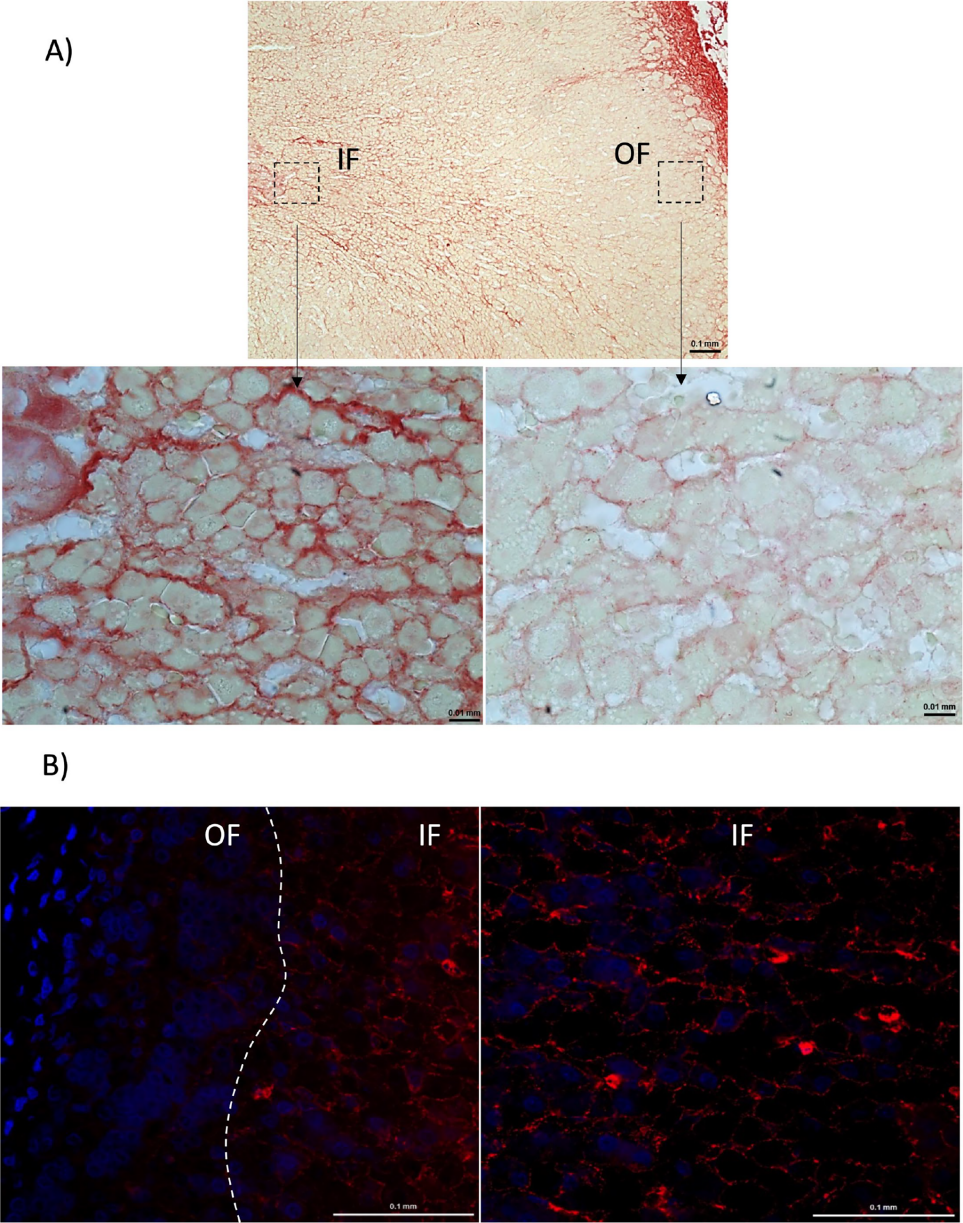
Validation of ECM protein expression in the porcine adrenal cortex. (A) Representative images of Picrosirius Red staining for Collagen 1 (Col1). (B) Representative images of immunohistochemistry for Collagen VI (Col6). OF = outer adrenal fraction; IF = inner fraction.

As a limitation of the study design, we can highlight the number of grouped samples, which, for ethical reasons, restricted the number of animals sacrificed and the decellularization protocol. Cellular remnants after decellularization could interfere with proteomic analysis by creating background noise or masking low-abundance proteins. During decellularization, the proteins may undergo modifications that alter the protein structure and function, making it challenging to identify and characterize them accurately via proteomic analysis. Additionally, proteomic techniques may have limited sensitivity in detecting low-abundance proteins. Furthermore, the proteomic data analysis from decellularized tissues is complex due to the large volume of generated data. To address this, integrating proteomics data with other data sets, such as transcriptomics, can provide a more comprehensive understanding of tissue composition and function.

## Conclusions

Our study represents the first comparative analysis of the ECM-specific proteome of the outer and inner fractions of the porcine adrenal cortex. The components and related ECM proteins of OF and IF of the adrenal cortex demonstrate their distinct compositions.

Unlike the rat and human adrenal cortex, the inner fraction of the porcine adrenal cortex contains a richer and more complex array of proteins. This discovery warrants further investigation, as the porcine biomedical model can be valuable for translational medical research.

## Supplementary Material









## Notes

(Figure S1) Certification of decellularization of adrenal fraction sample; (Figure S2) porcine adrenal gland; and (Figure S3) overview of dimensional analyses and protein contributions (PDF)
List of peptides in which proteins were identified (XLSX)
Total proteins identified in the porcine adrenal cortex’s outer (OF) and inner (IF) fractions (XLSX)
Extracellular matrix (ECM) proteins identified in the porcine adrenal cortex’s outer (OF) and inner (IF) fractions (XLSX)
