# Supplementary material for: Proteomic Analysis of the Extracellular Matrix in the Porcine Adrenal Cortex
Source: J Proteome Res. 2025 Jun 10;24(7):3300–9. doi: 10.1021/acs.jproteome.5c00026 (PMC12235688; doi:10.1021/acs.jproteome.5c00026)
Supplement: Supplementary file 1 [file pr5c00026_si_001.pdf]

Proteomic analysis of the extracellular matrix in the porcine adrenal cortex.

Jean Lucas Kremer[1], Henrique Sanchez Ortega[1], Talita Souza-Siqueira[3], Claudia Blanes Angeli[2], Leo Kei Iwai[4], Giuseppe Palmisano[2,5], Claudimara Ferini Pacicco Lotfi[1*]

1. Institute of Biomedical Sciences, Department of Anatomy, University of São Paulo, São Paulo, Brazil, Av. Prof. Lineu Prestes, 2415, Butantã, São Paulo, SP, 05508-000.
2. Institute of Biomedical Sciences, Department of Parasitology, University of São Paulo, Brazil, Av. Prof. Lineu Prestes, 1374, Butantã, São Paulo, SP, 05508-000.
3. School of Medicine, Department of Clinical Medicine, University of São Paulo, Brazil, Av. Dr. Arnaldo, 455, Cerqueira César, São Paulo, SP, 01246903
4. Laboratory of Applied Toxinology, Center of Toxins, Immune-response and Cell Signaling LETA/CeTICS, Butantan Institute, Brazil, Av. Vital Brasil, 1500 - Butantã, São Paulo, SP, 05503-900.
5. School of Natural Science, Macquarie University, Australia, Angel Place, 123 Pitt Street, Sydney, NSW 2109.
*Corresponding Author: clotfi@usp.br

List of supplementary materials

Supplementary Figure S1: Certification of decellularization of adrenal fraction samples.
Supplementary Figure S2: The porcine adrenal gland.
Supplementary Figure S3: Overview of dimensional analyses and protein contributions.
Supplementary Table S1: The list of peptides in which proteins were identified.
Supplementary Table S2: Total proteins identified in the porcine adrenal cortex's outer (OF) and inner (IF) fractions.
Supplementary Table S3: Extracellular Matrix (ECM) proteins identified in the porcine adrenal cortex's outer (OF) and inner (IF) fractions.

A)

Quantification of DNA from control and decellularized of porcine adrenal gland fractions (ng/µl)

Sample	Outer Fraction (OF)	Inner Fraction (IF)
Control	1024.9 ± 67.4	982.6 ± 58.6
1	41.2 ± 5.2	37.4 ± 2.9
2	46.5 ± 9.6	23.6 ± 4.6
3	42.5 ± 7.0	34.5 ± 2.8
4	43.9 ± 5.2	26.2 ± 4.0
5	45.1 ± 7.1	47.2 ± 2.6

B)

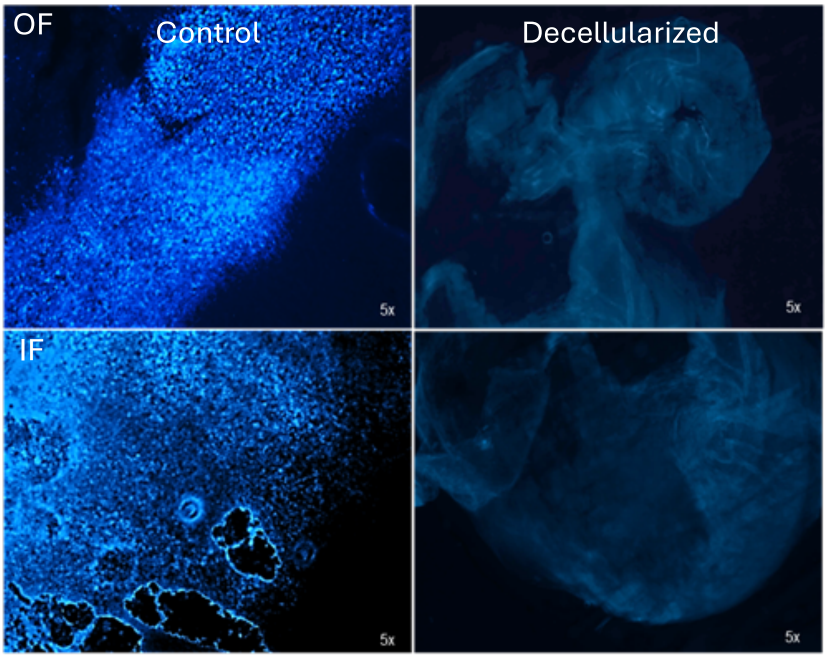

Figure S1 – Certification of decellularization of adrenal fraction samples. A) DNA quantification; B) Staining with DAPI (4',6-diamidino-2-phenylindole).

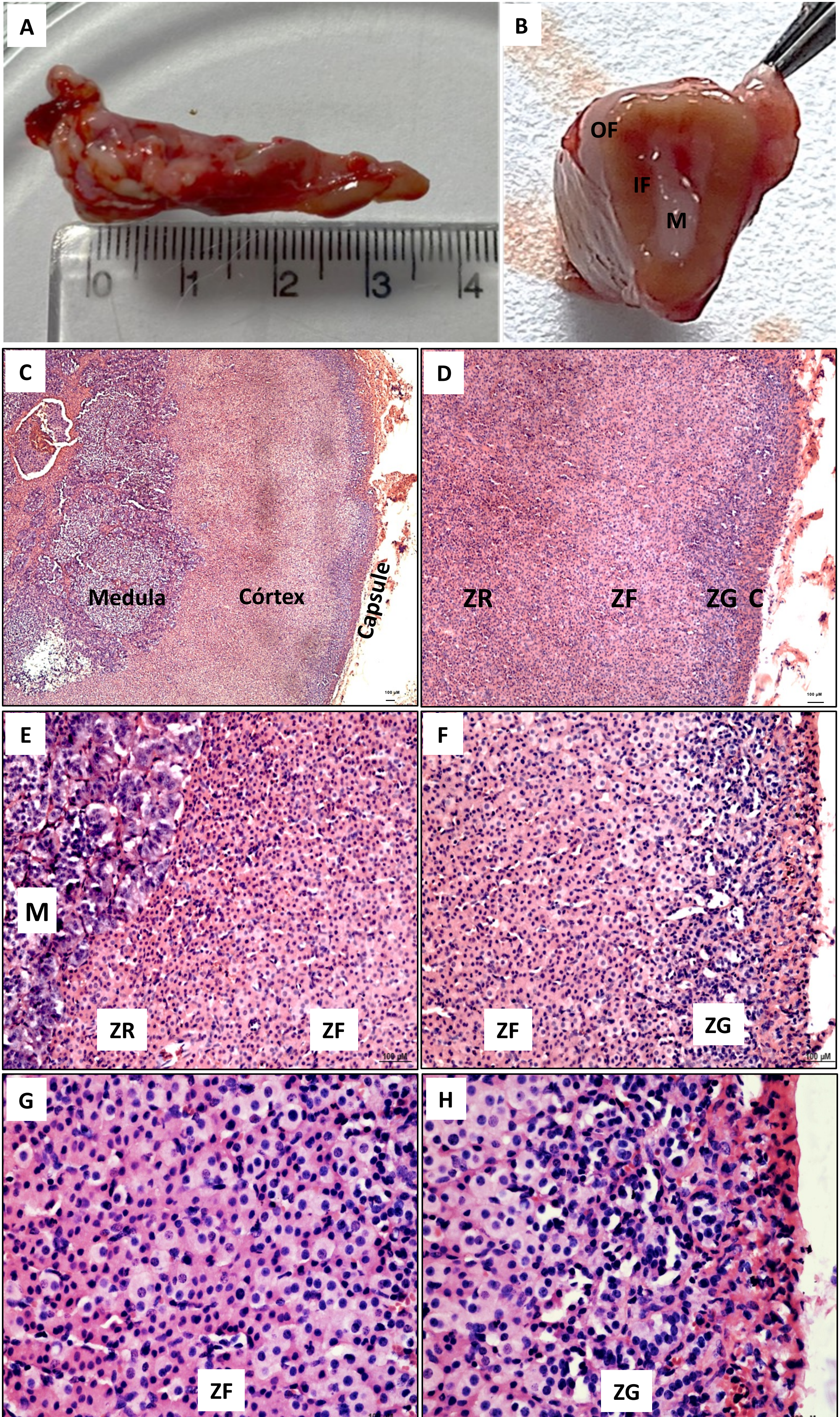

Figure S2. The porcine adrenal gland. A) the entire adrenal gland B) the transverse section of the adrenal gland; C-H) Adrenal gland sections at different magnifications stained with Hematoxylin and Eosin. OF= outer fraction (capsule + ZG); IF=inner fraction (ZF + ZR); M=medulla; C=capsule; ZG=zone glomerulosa; ZF=zone fasciculata; ZR=zone reticularis.

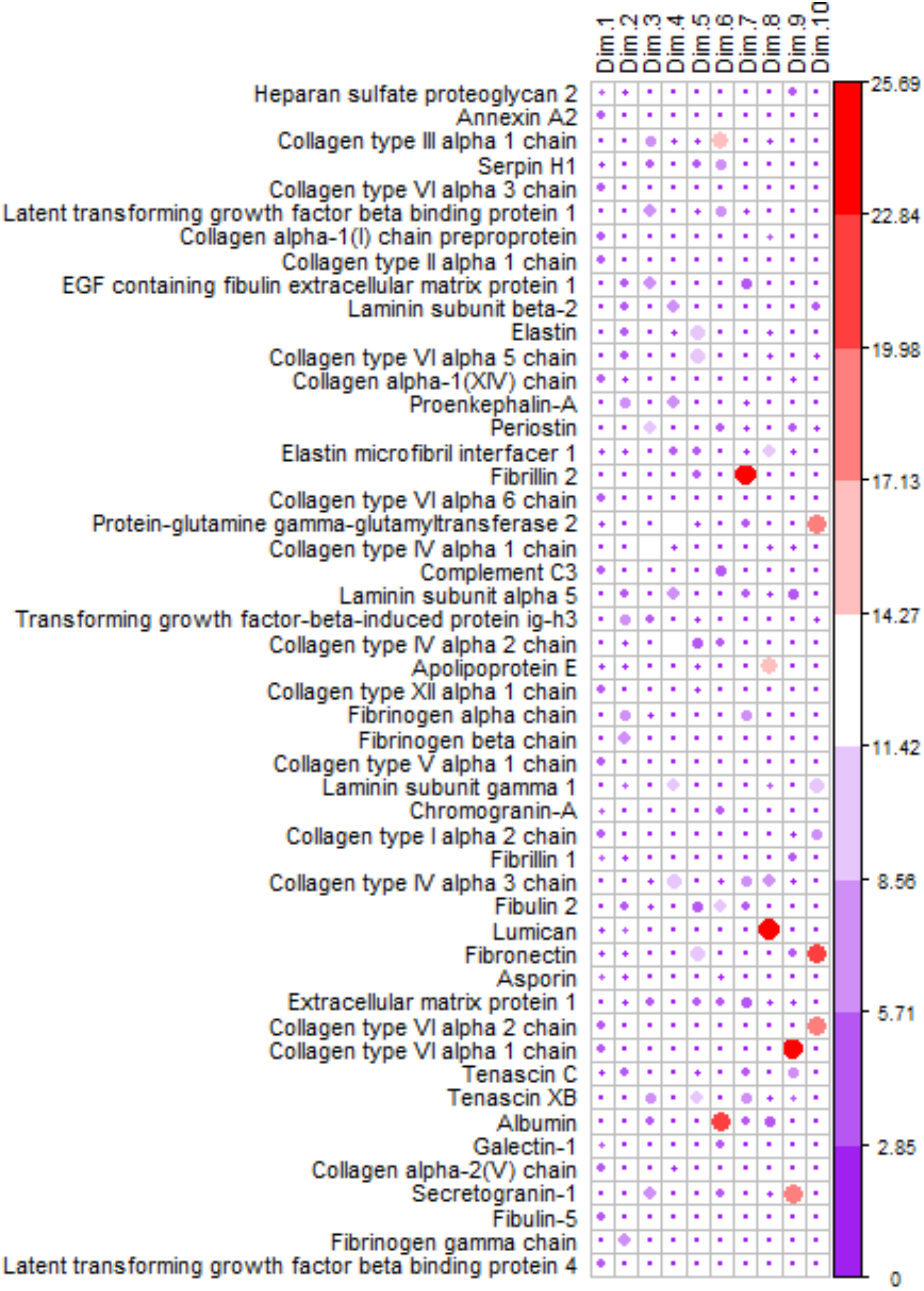

Figure S3. Overview of dimensional analyses and protein contributions. The graph illustrates the distribution of proteins about each dimension, with the color gradient and symbol size representing the percentage contribution of each protein in its corresponding dimension, ranging from 0 to 40.19%. Generated by R software, version R-4.3.3.
